# Isolation and Expression of Glucosinolate Synthesis Genes *CYP83A1* and *CYP83B1* in Pak Choi (*Brassica rapa* L. ssp. *chinensis* var. *communis* (N. Tsen & S.H. Lee) Hanelt)

**DOI:** 10.3390/ijms13055832

**Published:** 2012-05-15

**Authors:** Biao Zhu, Zhizhou Wang, Jing Yang, Zhujun Zhu, Huasen Wang

**Affiliations:** 1 Department of Horticulture, Zhejiang University, Zijingang Campus, Yuhangtang Road 866, Hangzhou 310058, China; E-Mails: billzhu@zju.edu.cn (B.Z.); twzz@163.com (Z.W.); 2 Department of Horticulture, School of Agricultural and Food Science, Zhejiang A & F University, Huan Cheng Bei Lu 88, Lin’an, Hangzhou 311300, China; E-Mails: yangjing@zafu.edu.cn (J.Y.); whsych66@163.com (H.W.)

**Keywords:** pak choi, glucosinolate, phylogenetic analysis, quantitative real-time PCR

## Abstract

*CYP83A1* and *CYP83B1* are two key synthesis genes in the glucosinolate biosynthesis pathway. *CYP83A1* mainly metabolizes the aliphatic oximes to form aliphatic glucosinolate and *CYP83B1* mostly catalyzes aromatic oximes to synthesis corresponding substrates for aromatic and indolic glucosinolates. In this study, two *CYP83A1* genes named *BcCYP83A1-1* (JQ289997), *BcCYP83A1-2* (JQ289996) respectively and one *CYP83B1* (*BcCYP83B1*, HM347235) gene were cloned from the leaves of pak choi (*Brassica rapa* L. ssp. *chinensis* var. *communis* (N. Tsen & S.H. Lee) Hanelt) “Hangzhou You Dong Er” cultivar. Their ORFs were 1506, 1509 and 1500 bp in length, encoding 501, 502 and 499 amino acids, respectively. The predicted amino acid sequences of *CYP83A1-1*, *CYP83A1-2* and *CYP83B1* shared high sequence identity of 87.65, 86.48 and 95.59% to the corresponding ones in *Arabidopsis*, and 98.80, 98.61 and 98.80% to the corresponding ones in *Brassica pekinensis* (Chinese cabbage), respectively. Quantitative real-time PCR analysis indicated that both *CYP83A1* and *CYP83B1* expressed in roots, leaves and petioles of pak choi, while the transcript abundances of *CYP83A1* were higher in leaves than in petioles and roots, whereas *CYP83B1* showed higher abundances in roots. The expression levels of glucosinolate biosynthetic genes were consistent with the glucosinolate profile accumulation in shoots of seven cultivars and three organs. The isolation and characterization of the glucosinolate synthesis genes in pak choi would promote the way for further development of agronomic traits via genetic engineering.

## 1. Introduction

Pak choi (*Brassica rapa* L. ssp. *chinensis* var. *communis* (N. Tsen & S.H. Lee) Hanelt) is a very important vegetable crop in China and Northeast Asia because of its high yield and nutritional value [1]. It contains a number of bioactive components such as folates, vitamin C, carotenoids, polyphenols and glucosinolates which have anti-carcinogenic effects [2–4]. Glucosinolates are a group of plant secondary metabolites derived from different kinds of amino acids and can be hydrolyzed by myrosinase to produce a variety of compounds which can have beneficial effects on human health including anti-carcinogenic, cholesterol-reducing and other pharmacological effects [5]. The glucosinolates-myrosinase-system is also considered to be an efficient defense system for plant against herbivorous generalists and microorganisms [6–8].

To date, more than 132 individual glucosinolates have been detected, and they are grouped into aliphatic, aromatic and indole glucosinolates depending on the structure of their side-chain [9]. In recent years, significant advances have been made in our understanding of the biosynthetic pathway of glucosinolates. The formation of glucosinolates can be divided into three separate phases including the chain elongation, formation of the core structure and secondary modifications of side chain and glucose moiety of the parent glucosinolates [10]. Since the completion of the Arabidopsis genome sequencing, a significant progress on glucosinolate research has been made especially in the identification of the genes and regulators for glucosinolate biosynthesis. MAM (Methylthioalkylmalate synthase) genes are responsible for the chain-elongation. CYP79 enzymes catalyze the first committed step of glucosinolates core structure formation as the conversion of amino acids to aldoximes. CYP83 enzymes catalyze the key step for the core structure formation as the conversion of aromatic oximes, and the conversion of thiohydroximic acids to glucosinolates [11–18].

Of the two oxime-metabolizing enzymes CYP83A1 and CYP83B1, major progress has been made by using *Arabidopsis* as model plant. Aliphatic oximes derived from chain-elongated homologs of methionine are efficiently metabolized by CYP83A1 enzyme, whereas CYP83B1 enzyme metabolizes these substrates with very low efficiency. Aromatic oximes derived from phenylalanine, tryptophan, and tyrosine are metabolized by both enzymes, although CYP83B1 has higher affinity for these substrates than CYP83A1 [19,20]. However, most of these researches are carried out in the model plant *Arabidopsis*. As glucosinolates have been proved to have many beneficial effects on human health and *Arabidopsis* is not edible, it is necessary to focus on glucosinolates-containing crops which are important for human consumption. In the present study, two glucosinolates biosynthesis genes *BcCYP83A1* and *BcCYP83B1* were isolated from pak choi and characterized and their expression pattern in different organs and cultivars were also characterized.

## 2. Results and Discussion

### 2.1. Identification and Sequence Analysis of CYP83A1 and CYP83B1

**Figure 1:**
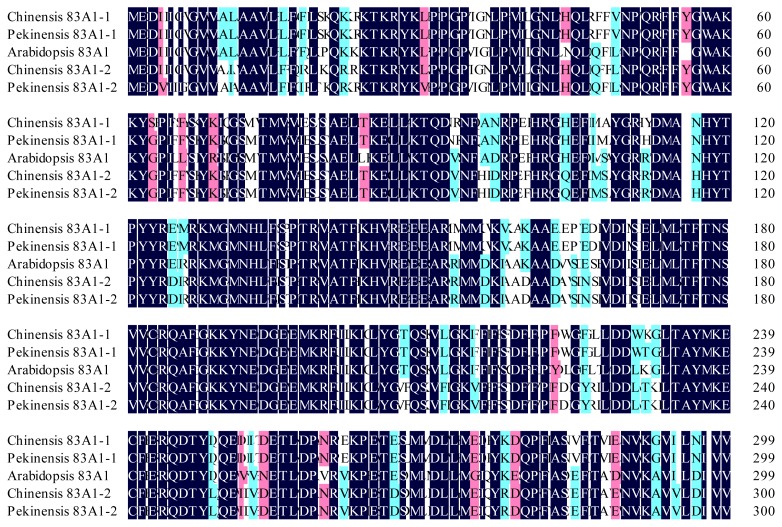

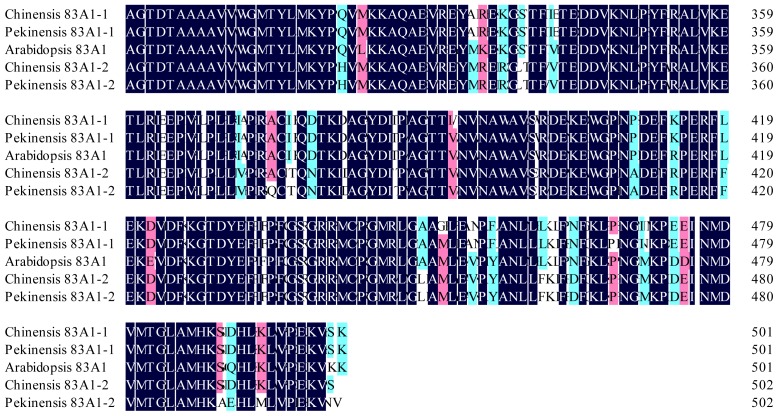
Multiple alignment of deduced amino acid sequences of *CYP83A1*. Black shaded and other shaded boxes show identical and similar amino acids, respectively. GenBank accession number of the *CYP83A1s: Arabidopsis thaliana* (NM117451), *Brassica pekinensis* (FJ376049, FJ376050) and *Brassica chinensis* (JQ289997, JQ289996).

**Figure 2:**
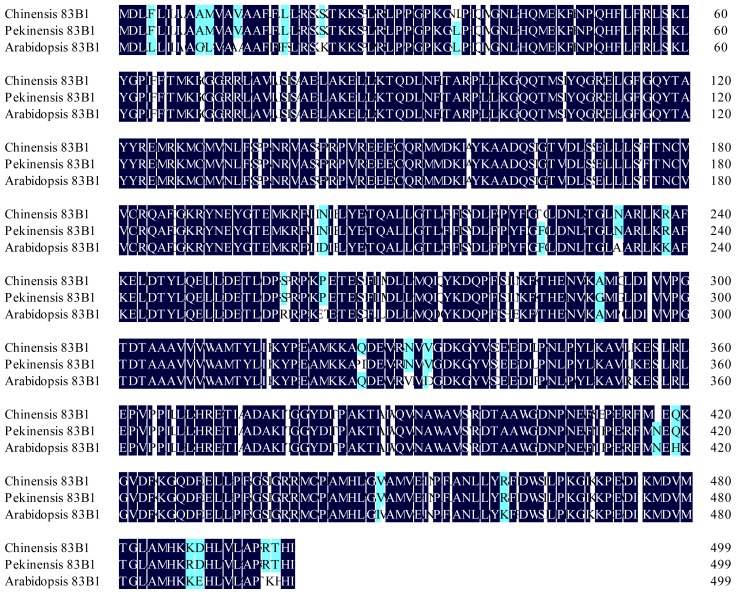
Multiple alignment of deduced amino acid sequences of *CYP83B1*. Black shaded and other shaded boxes show identical and similar amino acids, respectively. GenBank accession number of the *CYP83B1s: Arabidopsis thaliana* (NM119299), *Brassica pekinensis* (FJ376051) and *Brassica chinensis* (HM347235).

Two aliphatic oxime-metabolizing enzymes namely *BcCYP83A1-1*, *BcCYP83A1-2*, and one aromatic oxime-metabolizing enzyme *BcCYP83B1* were isolated from the leaves of pak choi “Hangzhou You Dong Er” cultivar. The ORF of the genes were 1506, 1509 and 1500 bp in length, encoding 501, 502 and 499 amino acids, respectively (Figures 1 and 2). A BLAST search revealed that the primary structure of *CYP83A1-1*, *CYP83A1-2* and *CYP83B1* shared high sequence identity of 86.81, 87.43 and 90.13% to the corresponding ones in *Arabidopsis* (NM117451, NM119299), respectively, and shared high sequence identity of 99.14, 99.07 and 99.47% to the corresponding ones in *Brassica pekinensis* (*Brassica rapa* L. ssp. *pekinensis* var. *communis*, Chinese cabbage, FJ376049, FJ376050, FJ376051) respectively. The amino acid sequence deduced from *CYP83A1-1* and *CYP83A1-2* showed 87.65 and 86.48% identity to that of the *Arabidopsis CYP83A1*, and 98.80 and 98.61 % identity to *Brassica pekinensis CYP83A1-1* and *CYP83A1-2*, respectively. The deduced amino acid sequence of *CYP83B1* showed 95.59% and 98.80% identity to that of the *Arabidopsis* and *Brassica pekinensis*, respectively. The sequence similarity between *CYP83A1-1* and *CYP83A1-2* were 85.88% in this study, identical to that of 85.29% in *Brassica pekinensis*.

### 2.2. Phylogenetic Analysis of *CYP83A1* and *CYP83B1*

**Figure 3:**
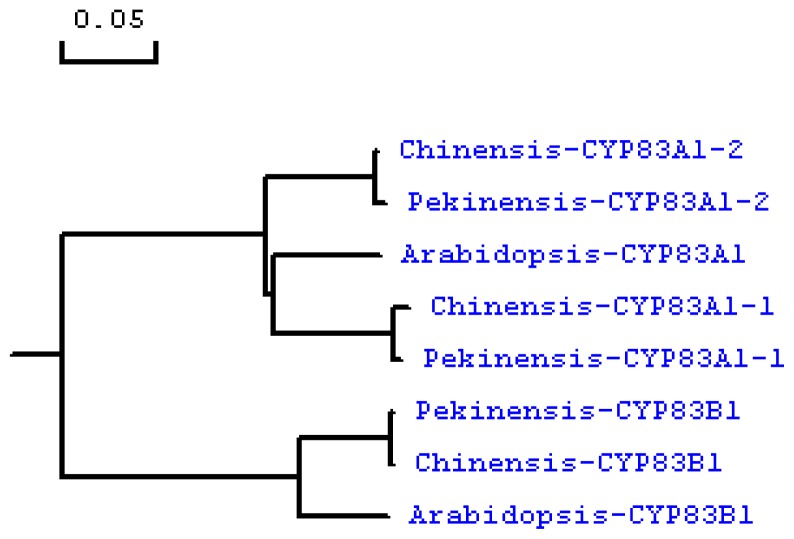
Phylogenetic tree of the cDNA sequences of the plant glycosyltransferase superfamily. GenBank accession numbers are: *Arabidopsis thaliana CYP83A1* (NM117451), *Arabidopsis thaliana CYP83B1* (NM119299), *Brassica pekinensis CYP83A1-1* and *CYP83A1-2* (FJ376049, FJ376050), *Brassica pekinensis CYP83B1* (FJ376051), *Brassica chinensis CYP83A1-1* and *CYP83A1-2* (JQ289997, JQ289996), *Brassica chinensis CYP83B1* (HM347235). The horizontal scale shows the number of differences per 100 residues derived from the ClustalW alignment.

We compared the isolated sequences with the corresponding ones in *Arabidopsis thaliana* (NM117451, NM119299) and *Brassica pekinensis* (FJ376049, FJ376050, FJ376051) by multiple alignments, and reconstructed the phylogenetic tree by ClustalW analysis program. The results showed that the homologs of *CYP83A1* and *CYP83B1* from the *Brassicaceae* family are highly conserved, with low variation in DNA sequence. As shown in the phylogenetic tree, the glucosinolate synthesis genes of *Brassica chinensis* and *Brassica pekinensis* clustered first with each other and then with the genes of *Arabidopsis* (Figure 3), which demonstrated the highly congruent relationship among different sources of glucosinolate synthesis genes.

### 2.3. Expression of *CYP83A1* and *CYP83B1* in Different Cultivars and Various Organs

**Figure 4:**
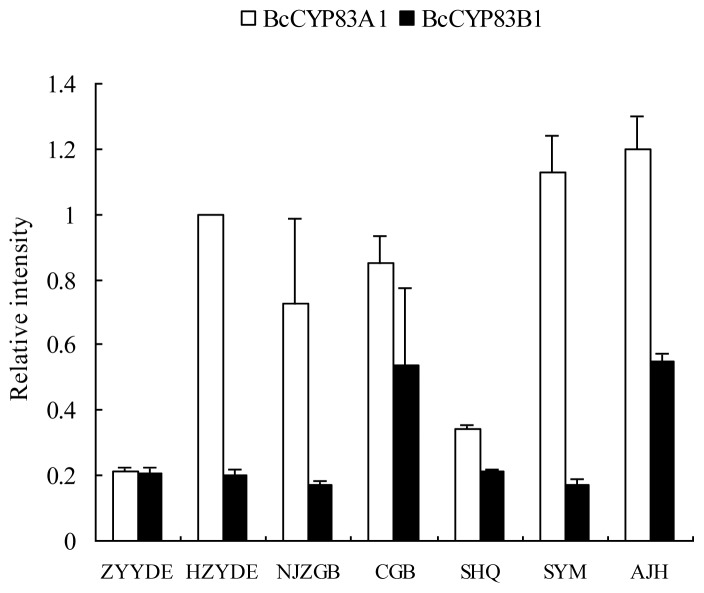
Relative expression analysis of *CYP83A1* and *CYP83B1* in leaves of seven cultivars of pak choi. Error bars represent the standard deviation (SD) from three biological repeats.

Quantitative real-time PCR (qRT-PCR) was employed to confirm the expression patterns of the two genes in seven cultivars, as well as in different organs. Actin was used as an internal reference control for total RNA input. In our analysis, the gene-specific primers used for amplification of *CYP83A1* recognized and amplified both *CYP83A1-1* and *CYP83A1-2* genes. Both *CYP83A1* and *CYP83B1* were detected in the leaves of all the seven cultivars and had the same expression pattern except for cultivar “ZYYDE”. The expression patterns of the two genes in leaves of seven cultivars are illustrated in Figure 4. *CYP83A1* was presented in higher abundance compared with *CYP83B1* in most of the cultivars, whereas the *CYP83A1* and *CYP83B1* transcripts in “ZYYDE” cultivar were nearly same.

**Figure 5:**
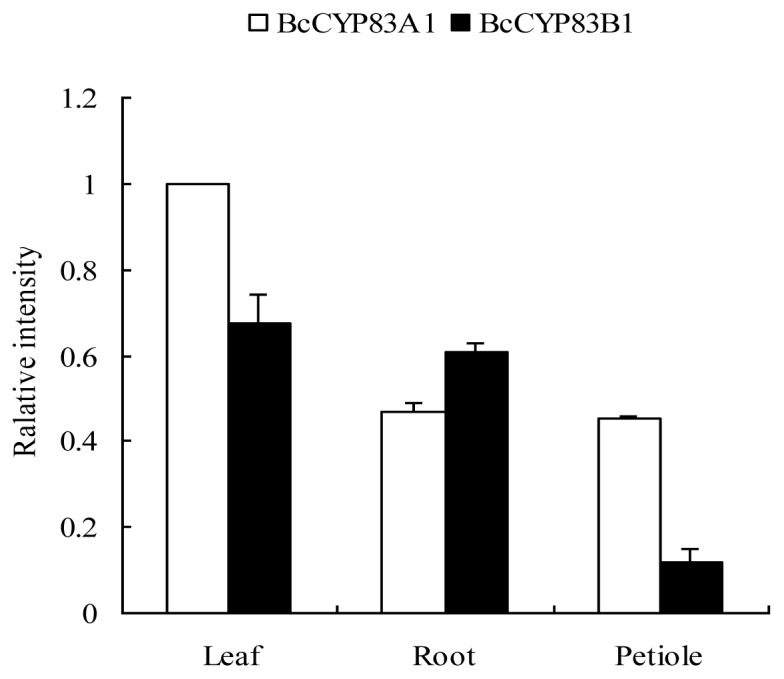
Relative expression analysis of *CYP83A1* and *CYP83B1* in leaf, petiole and root of pak choi cv. HZYDE. Error bars represent the standard deviation (SD) from three biological repeats.

Both the aliphatic oxime-metabolizing enzyme and aromatic oxime-metabolizing enzyme were identified in the three organs (roots, leaves and petioles) of pak choi (cv. HZYDE). Though *CYP83A1* and *CYP83B1* exist in all those organs, they displayed spatial expression patterns (Figure 5). The expression level of *CYP83A1* was higher in leaves than in roots or petioles, whereas the *CYP83B1* showed higher mRNA abundance in roots compared with another two.

### 2.4. Glucosinolate Profile Concentrations in Different Cultivars and Various Organs

The concentrations of aliphatic, indole and aromatic glucosinolates in different pak choi cultivars and in different organs of “Hangzhou You Dong Er” cultivar were analyzed. As shown in Table 1, the aliphatic glucosinolate concentrations in leaves of most of the cultivars were higher than indole and aromatic glucosinolates. The glucosinolate profiles in different organs varied differently. As leaf and petiole had particularly higher aliphatic glucosinolates, however, root had much higher indole and aromatic glucosinolates.

### 2.5. Discussion

In this study, we isolated two *CYP83A1* genes and one *CYP83B1* gene from pak choi “Hangzhou You Dong Er” cultivar. Amino acid sequence analysis and sequence alignment with their homologs from *Brassica pekinensis* and *Arabidopsis* showed the two key glucosinolate synthetic genes *CYP83A1* and *CYP83B1* within *Brassicaceae* family are highly conserved. As shown in Figures 1 and 2, the genes isolated from pak choi have both high DNA and amino acid sequence conservation with their *Brassica pekinensis* counterparts. The identity of DNA sequence and deduced amino acid sequence of *CYP83B1* between the two species was 99.47% and 98.80%. The two copies of *CYP83A1* namely *BcCYP83A1-1* and *BcCYP83A1-2* from pak choi were also highly similar to the corresponding ones of *Brassica pekinensis*, whereas there was only one copy of *CYP83A1* in *Arabidopsis*. This is consistent with the previous finding by Zang *et al.* that identities within *Brassica rapa* were usually higher than those with *Arabidopsis* [21]. They isolated and analysed the glucosinolate synthesis genes in *Brassica pekinensis* by genome-wide identification [21]. The result was also accordant to the notion that *Brassica* species evolved and were triplicated after divergence from *Arabidopsis* lineage [22–24]. The observed glucosinolate synthesis sequence similarities among the *Brassicas* (mostly >90%) were higher than those between *Brassica* and *Arabidopsis* (mostly 80–90%) in this study also supported the presumptive evolution order.

Consistent with the results of previous studies in *Arabidopsis*, both the *CYP83B1* and *CYP83A1* transcripts were expressed in all tested organs (roots, leaves and petioles) of pak choi [25,26]. Mizutani reported that *CYP83A1* expression levels in *Arabidopsis* was highest in leaves and significantly higher in roots and stems than in flowers and siliques, while *CYP83B1* was the most highly expressed in roots [25]. In our study, the expression level of *CYP83A1* was also highest in leaves, and the *CYP83B1* expressed higher in leaves and roots than in petioles. These results were consistent with the previous finding that *CYP83A1* expressed highest in the leaves and *CYP83B1* were preferentially expressed in the roots [25,26]. The gene expression pattern in the organs was consistent with the glucosinolate profile accumulations as aliphatic glucosinolates were higher in leaves and petioles while indole and aromatic glucosinolates were higher in roots. The results were similar to Brown’s finding that aliphatic glucosinolates are typically higher in the leaves compared to the roots while indole glucosinolates are abundant in the roots [27]. When compared within an appointed organ, we found that the expression patterns of the two genes were different. *CYP83A1* transcripts were almost two-folds of *CYP83B1* transcripts in leaves and petioles, in contrast, the expression levels of the *CYP83A1* were relatively lower than *CYP83B1* in roots. Our finding was also accordant to the conclusion that the CYP83A1 catalyzed aliphatic glucosinolates biosynthesis and accumulation in leaves and the CYP83B1 catalyzed aromatic and indole glucosinolates mostly found in roots [15].

The expression patterns of the two genes in leaves of seven cultivars in our study were similar except “ZYYDE” cultivar and the gene expression levels were consistent with the glucosinolate profile accumulation. The expression levels of *CYP83A1* in leaves of six of seven cultivars were higher than *CYP83B1* and the aliphatic glucosinolates of the six *CYP83A1* higher expression cultivars were higher than another two glucosinolate profiles, whereas *CYP83A1* and *CYP83B1* expressed nearly identical in “ZYYDE” cultivar. While focused on the specific gene expression and glucosinolate accumulation in individual cultivar, “SYM” cultivar had high *CYP83A1*/*CYP83B1* transcript level ratio and high aliphatic glucosinolates/indole glucosinolates ratio. And “CGB” cultivar had the higher *CYP83B1* transcript level and relative higher aromatic glucosinolates accumulation.

## 3. Experimental Section

### 3.1. Plant Materials

The seeds of seven pak choi cultivars (*Brassica rapa* L. ssp*. chinensis* var. *communis* (N. Tsen & S.H. Lee) Hanelt) with different characters were sown in plastic pot with vermiculite in a growth chamber with 26/22 °C (12 h light/12 h dark) for 2 weeks. The seven commonly plant and consumed pak choi cultivars in southern China used are “Hangzhou You Dong Er” (HZYDE), “Zhou Ye You Dong Er” (ZYYDE), “Si Yue Man” (SYM), “Shanghai Qing” (SHQ), “Nanjing Zhong Gan Bai” (NJZGB), “Chang Geng Bai” (CGB) and “Ai Jiao Huang” (AJH). The young leaves from two-week old plants of seven pak choi cultivars were collected as experimental materials for quantitative real-time PCR (qRT-PCR) analysis. And three organs including roots, petioles and leaves of “Hangzhou You Dong Er” cultivar were collected for the expression analysis.

### 3.2. Isolation of Total RNA and Synthesis of Full-Length cDNA Sequence

Total RNA was extracted from samples using TRIzol reagent RNAisoTM Plus (Takara, D9108A) following the manufacturer’s protocols. Approximately 0.1 g of tissue was extracted in 1 mL of extraction buffer. After melted in RNA-free water, RNA was quantified by UV spectroscopy and its integrity was visually assessed on ethidium bromide stained agarose gels. The cDNA was synthesized from 500 ng of DNA-free RNA with a PrimeScript RT reagent Kit (Takara, DRR037A) following the manufacturer’s protocol.

Degenerate primers allowing amplification were designed based on sequences corresponding to highly conserved peptide regions of *CYP83A1* and *CYP83B1* of *Arabidopsis* and *Brassica pekinensis*. The cDNAs were amplified by PCR using the degenerate primers, then cloned into the pMD18-T Easy vector (Takara, Code D101A) and sequenced (Invitrogen). Rapid amplification of cDNA ends (RACE) was performed to obtain the 3′ and 5′ ends of the genes with the SMART RACE cDNA Amplification Kit (Clontech) according to the manufacturer’s instructions. Sequence analysis of open reading fragment (ORF) and encoded amino acid sequence of genes were deduced by BioXM 2.6 (Msight, Guangzhou, China, 2007). Physicochemical properties of the deduced protein were predicted by Protparam [28].

### 3.3. Phylogenetic Analysis

The nucleotide and deduced amino acid sequences were used for multiple alignments and phylogenetic tree analysis. The tree was obtained by ClustalW analysis program [29].

### 3.4. Quantitative Real-Time RT-PCR

The oligonucleotide primers for quantitative real-time PCR (qRT-PCR) analysis were designed with primer3 (version 0.4.0) based on the gene sequences (JQ289997, JQ289996, HM347235 and EX087730) [30]. For *CYP83A1*, we designed a specific primer which can amplify both *CYP83A1-1* and *CYP83A1-2* for qRT-PCR according to the highly conserved peptide regions compared with *Brassica chinensis*, *Brassica pekinensis* and *Arabidopsis*. The qRT-PCR primers for *CYP83B1* and actin gene were designed the same way. The size of all qPCR products was limited to 100–300 nucleotides. PCR product size was analyzed on 1% agarose gels stained with ethidium bromide and the specificity of qRT-PCR products was then confirmed by sequencing. The qPCR primers of *CYP83A1*, *CYP83B1* and Actin were as follows respectively:

**Table:** 

*CYP83A1*
(F) 5′-TCCTCTCCTTATCCCTCGTGCTTGC-3′
(R) 5′-CCAAGACGCATTCCAGGGCACATTC-3′
*CYP83B1*
(F) 5′-AGACTCTTGACCCTAGCCGTCCTA-3′
(R) 5′-CCTTTGTCACCGACCACATTCCT-3′
*Actin*
(F) 5′-CGCTTAACCCGAAAGCTAAC-3′
(R) 5′-TACGCCCACTAGCGTAAAGA-3′

For gene expression analysis, first-stranded cDNAs from the leaves of the seven cultivars, and first-stranded cDNAs from the leaves, the petioles and the roots of “HZYDE” cultivar were used as templates. RT-PCR was performed on Applied Biosystems 7300 Real-Time PCR system with the SYBR Green qPCR kit (Takara, DRR081A). Ten-fold diluted cDNA was used as the template in real-time PCR. 25 μL total reaction volume comprised 12.5 μL 2× SYBR Green PCR Master Mix (Takara), 1 μL of each primer (10 μM), 2 μL of cDNA, and 8.5 μL of PCR-grade water. The PCR conditions were as the follows: initiated by 30 s at 95 °C, then followed by 40 cycles of 95 °C for 5 s, 60 °C for 31 s, and completed with a melting curve analysis program. No-template controls and melting curve analyses were included in every reaction. Brassica actin (EX087730) was used as the housekeeping gene. A melting curve of denatured double-stranded cDNA was established to test the purity of the products, and the products were re-sequenced to confirm the specificity. Triplicate quantitative PCR experiments were performed for each sample, and the expression values obtained were normalized against actin. Analysis of the relative gene expression data was done using the 2^−ΔΔCt^ method [31,32].

### 3.5. Glucosinolate Analysis

The glucosinolate extraction and analysis procedure was performed according to the method of Krumbein with slight modification [33]. Briefly, 0.25 g sample powder were boiled with 10 mL 70% methanol to inactive myrosinase, then the supernatants were loaded onto a 1 mL mini-column (JT Baker, Phillipsburg, NJ, USA) containing 500 μL activated DEAE Sephadex™ A25 (Amersham Biosciences, Uppsala, Sweden) to de-sulphate overnight with aryl sulfatase (Sigma-Aldrich Co., St. Louis, MO, USA). The resultant desulpho (ds)-glucosinolates were eluted with ultra pure water and stored at −20 °C prior to separation by high performance liquid chromatography (HPLC).

For HPLC, 20 μL samples were analyzed in an Agilent 1200 HPLC system (G1311A quaternary pump, G1322A vacuum solvent delivery degasser, G1316A thermostatted column compartment, Agilent Technologies, Inc., Palo Alto, CA, USA) consisting of a G1329A auto injector, a prontosil ODS2 column (250 × 4 μm, 5 μm, Bischoff, Leonberg, Germany) and a G1315B diode array detector (DAD) set at 229 nm. The mobile phase was ultra pure water (A) and acetonitrile (Tedia, Fairfield, OH, USA) (B) in a linear gradient from 0% to 20% B in 32 min, then constant 20% B for 6 min, following by 100% B and 0% B prior to the injection of the next sample. The flow rate was 1.3 mL·min^−1^.

## 4. Conclusions

In the present study, we isolated and analyzed the glucosinolate biosynthetic genes in pak choi. This is the first report about glucosinolate genes in pak choi although they have been studied in *Arabidopsis*. The gene expression levels and glucosinolate profile accumulation were also compared. The isolation and further characterization of the genes responsible for the synthesis and regulation of glucosinolates in pak choi are important for the development of vegetables that are beneficial to health.

## Figures and Tables

**Table 1:** Aliphatic, indole and aromatic glucosinolates content (μmol) in shoots of different cultivars and in different organs of “Hangzhou You Dong Er” cultivar. Data are the means of three biological repeats ± standard deviation.

	ZYYDE	HZYDE	NJZGB	CGB	SHQ	SYM	AJH	Leaf	Root	Petiole
Aliphatic GS	0.43 ± 0.07	0.94 ± 0.07	0.49 ± 0.07	0.54 ± 0.08	0.98 ± 0.04	2.90 ± 0.34	0.87 ± 0.10	1.89 ± 0.29	0.12 ± 0.02	0.48 ± 0.05
Indole GS	0.68 ± 0.03	0.76 ± 0.03	0.30 ± 0.01	0.45 ± 0.02	0.52 ± 0.02	0.59 ± 0.03	0.75 ± 0.10	0.25 ± 0.06	0.94 ± 0.10	0.24 ± 0.04
Aromatic GS	0.38 ± 0.02	0.51 ± 0.02	0.31 ± 0.03	0.59 ± 0.07	0.63 ± 0.04	0.46 ± 0.07	0.61 ± 0.05	0.28 ± 0.06	0.78 ± 0.07	0.25 ± 0.03
Total GS	1.49 ± 0.07	2.21 ± 0.09	1.10 ± 0.09	1.58 ± 0.11	2.13 ± 0.05	3.95 ± 0.41	2.23 ± 0.04	2.43 ± 0.24	1.84 ± 0.17	0.97 ± 0.05
